# Practitioner Review: Infant mental health meets cell and molecular biology – a look to the future

**DOI:** 10.1111/jcpp.70097

**Published:** 2026-01-07

**Authors:** Charles H. Zeanah, Megan Hare, Katherine Cowhey, Stacy S. Drury

**Affiliations:** ^1^ Tulane University School of Medicine New Orleans LA USA; ^2^ Boston Children's Hospital Harvard Medical School Boston MA USA

**Keywords:** Early life experience, biology

## Abstract

**Background:**

A major research effort in the past two decades has begun to illuminate how experience ‘gets under the skin’ – that is – the cellular and molecular processes that are associated with adversity and resilience.

**Methods:**

We selectively review three areas of this research: epigenetics, especially DNA methylation, telomere length, and inflammatory processes, and consider the implications of this work for better understanding the effects of adversity and pathways of recovery.

**Results:**

Because infant mental health practitioners focus on children in the earliest years of life, they are well positioned to favorably alter the developmental trajectories of children experiencing or at risk for maladaptation. In addition to helping us develop more individually effective treatments, we consider other ways in which research advances in cell and molecular biology may be especially important to infant mental health practitioners in the future.

**Conclusions:**

Better understanding these processes will enhance effectiveness and potentially enlarge the scope of our practice.

## Introduction

The dominant model of developmental psychopathology in the last quarter century has been the cumulative risk model, which holds that the number rather than the nature of risk factors is the best predictor of adverse outcomes. The influential Adverse Childhood Experiences (ACE) study (Felitti et al., 1998), for example, demonstrated that an increasing number of types of adversity predicted adverse outcomes – even without consideration of severity, timing of exposure, or chronicity. One of the more notable features of this study is its demonstration that a large number of health – not just mental health – outcomes in adulthood were associated with the number of adverse experiences in childhood. It was quite surprising and novel that diseases such as ischemic heart disease, cancer, chronic lung disease, skeletal fractures, and liver disease were associated with the number of types of adversity in childhood (Felitti et al., 1998). Furthermore, an individual with an ACE score (total of 10 possible types of childhood adversity) of 6 or greater had a 20‐year reduction in life expectancy (Brown et al., 2009). Despite the retrospective design and the relatively homogenous population of this first seminal study, the findings of associations between a range of different physical and mental health disorders and diseases have been replicated repeatedly (Madigan et al., 2023), including in young children (e.g., Kerker et al., 2015). More recently, it has become clear that the association between early life adversity and poor health in children and adults is not mediated only through health risk behaviors, but also through activation of cellular and molecular pathways related to health and disease.

The ACE study, along with research examining additional adversities such as neighborhood dysfunction, racism, and exposure to community violence, has inspired scholars across various disciplines to explore the mechanisms by which early life adversity, particularly within the family context, contributes to the development of disease – what is often referred to as ‘how experience gets under the skin’ (Hertzman & Boyce, 2010) or ‘biological embedding’ (Hertzman, 2012). Ongoing research into the mechanisms of biological embedding spans neurodevelopmental and cognitive processes, brain function, physiological responses, and intricate cellular and molecular mechanisms. These efforts aim to elucidate how an individual's social environment can lead to a wide range of unwanted outcomes, including cardiovascular disease, obesity, smoking, academic achievement, accelerated aging, and memory decline (Hertzman & Boyce, 2010).

Research about biological embedding is growing, but much remains to be discovered. Ongoing advances in understanding the mechanisms of biological embedding are expected to shed light on the pathways leading from early adversity to negative health outcomes. Importantly, this research also holds the potential to identify pathways that promote protection, recovery, and resilience.

Specifically, the cellular and molecular processes involved in developing disease states may also underlie experiences promoting recovery and health. In addition, given the well‐documented importance of early experiences for brain development (Bick & Nelson, 2016), establishing and maintaining attachment relationships (Sroufe, Coffino, & Carlson, 2010), and the genesis of psychopathology (Sroufe, 1997), a better understanding of the cellular and molecular underpinnings of early experiences is a critical area of current and future clinical practice, research, and policy.

Because infant mental health is the clinical science of early experience (Zeanah & Zeanah, 2019), its practitioners are well positioned to intervene in the earliest years of life to promote healthy brain development, facilitate secure attachment relationships, and prevent or eliminate emerging psychopathology. Therefore, defining the processes of biological embedding is likely to become increasingly important as pathways and mechanisms are delineated. As early prevention and intervention efforts grow in number and scope, indicators of the efficacy of these interventions at the biological level will provide greater insight into pathophysiology and may well determine if our proposed interventions address not only the ‘surface scars’ of adversity seen in observable behavior and relationships but also address the biological traces intimately linked to later risk for physical and mental illness long after the exposure has ended. In this selective review, we illustrate several cellular and molecular processes relevant to early experience and their effects on development and the emergence of psychopathology. We conclude by exploring how these emerging findings may enhance clinical practice and inform interventions in the years to come.

### Epigenetics

Epigenetic changes mediate developmental processes, gene–environment interactions, and are relevant for activity‐dependent neuronal development (Karpova, Sales, & Joca, 2017). The best‐studied epigenetic markers, in relation to adversity, are DNA methylation (DNAm), with additional attention to histone modification. Epigenetic mechanisms primarily function to regulate differences in gene expression, typically by influencing mRNA levels without altering the underlying DNA sequence. These changes are a natural part of normative development and aging, shaped by genetic variation, life experiences, and environmental exposures. Dynamic epigenetic modifications are critical to human behavioral and emotional development, offering potential insights into how early exposures and experiences contribute to physiological changes and future risk for disorders.

Epigenetic changes occurring in childhood in response to harsh, inadequate, or unpredictable caregiving environments are of particular interest, as they may serve as biological indicators of future health risk, directly contribute to current psychopathology, and/or provide indication of successful treatment beyond behavioral symptom reporting. In this section, we discuss the most common epigenetic marker – DNA methylation – and its relevance to studies of early life adversity and across intergenerational transmission. It is important to acknowledge that while there are increasing data to support the relation between early life adversity and epigenetic changes, studies in humans typically measure DNAm in accessible biological samples, specifically saliva, buccal cells and peripheral blood, and rarely have used tissue derived from the central nervous system. As such, there is a presumption inherent in these studies of a high correlation of DNAm patterns (and other epigenetic factors, including telomere length) among peripheral tissues and between peripheral samples and expected patterns in the brain. Preclinical animal models do offer some support to these presumptions, as the majority of these are using brain tissue. Nevertheless, the cross‐tissue correlation of epigenetic markers and telomere length remains an area of active research, and future studies should carefully examine existing data to position sample collection to provide accurate and informative data (McLester‐Davis et al., 2023; Wolf et al., 2024).

### DNA methylation (DNAm)

DNAm is the most widely studied and best‐characterized epigenetic process. It describes adding a methyl group to a CpG dinucleotide (i.e., a cytosine base next to a guanine base), catalyzed by a family of enzymes called DNA methyltransferases (DNMT). DNMT1 is one of the most well‐studied enzymes controlling DNAm and has extensive expression in the brain and has been associated with schizophrenia, seizures, and cognitive delay (Boyce & Kobor, 2015). DNAm is critical for embryonic development, cellular differentiation, imprinting, and genome stability. The original canonical perspective of DNAm was that it occurred in key organizational areas of gene structure relevant to controlling expression levels, areas including promoter or enhancer, or through the recruitment of other molecules, including modifiers of histone acetylation. Typically, the greater the amount of DNAm at specific DNA sequence locations, the lower the amount of mRNA that is transcribed (converting the DNA sequence to an RNA strand) as a result of either interference with transcription factor (a protein that binds to a specific DNA sequence to assist in the creation of mRNA) binding or through chromatin (the tightly compacted DNA strand) structural remodeling.

Studies examining the impact of childhood maltreatment or early life adversity on DNAm have examined specific candidate genes, epigenome‐wide associations, and more recently epigenetic clocks, which are collections of DNAm sites across the genome thought to be related to different indicators of organismal, biological, and cellular aging (e.g., Horvath's clock). Of the gene‐specific studies, the majority have focused on genes involved in the regulation of the stress response systems, particularly the HPA axis and neuronal development (e.g., BDNF).

For example, a recent review identified that the most extensively studied genes related to epigenetic changes involve glucocorticoid signaling within the hypothalamic–pituitary–adrenal axis specifically, *NR3C1*, *FKBP5*, and the *CRH* gene family (Rubens et al., 2023). This system plays a crucial role in the body's stress response and is essential for understanding both health and disease.

### Building the evidence – early caregiving and epigenetic effects

The strongest evidence for the role of epigenetic changes related to early life experiences comes from a series of elegant experiments from Michael Meaney's laboratory and others. In these studies, it was shown that serotonin mediated the influence of maternal caregiving, specifically rodent licking and grooming, on the receptor to which cortisol binds, expressed through serotonin‐dependent increases in histone acetylation and promoter demethylation of the *Nr3c1* gene. Lower DNAm in the offspring of high licking and grooming mothers resulted in subsequent increased *Nr3c1* expression. Chemical blocking of demethylation, in the rodent model, prevented the downstream increase in Nr3c1 and associated behavioral differences in the offspring triggered by differences in the amount of maternal licking and grooming a pup received (Hellstrom, Dhir, Diorio, & Meaney, 2012; Weaver et al., 2004; Weaver, Diorio, & Meaney, 2007). Beyond the effect on *Nr3c1* and the HPA axis, this same measure of maternal care (licking and grooming) also was shown to influence hippocampal‐dependent neuronal plasticity. These effects occur through alterations in DNA methylation (DNAm) and histone acetylation of *Gad1*, a gene responsible for converting glutamate to GABA, a crucial inhibitory neurotransmitter (Zhang et al., 2016).

Many of these findings have been replicated in human studies; however, the directionality (i.e., increased or decreased) of alterations in DNA methylation levels and the relation to developmental psychopathology and physiologic regulation are mixed. This likely reflects the complex interplay between early experience, molecular pathways, and psychopathology. For example, hypermethylation of *NR3C1* has been reported in human studies of adults and children with a documented history of maltreatment (Perroud et al., 2011; Tyrka, Price, Marsit, Walters, & Carpenter, 2012). Increased DNAm in specific locations in the *NR3C1* gene was reported in cord blood from infants born to mothers with depression during the third trimester (Oberlander et al., 2008) and in postmortem brain tissue of suicide completers with a history of child abuse and decreased *NR3C1* mRNA expression (McGowan et al., 2009). Nevertheless, studies in adults with lifetime PTSD and a history of abuse reported decreased DNA methylation, suggesting that the long‐term epigenetic consequences of abuse may differ based on the timing of exposure, the timing of measurement of DNAm, and the presence of psychopathology, particularly PTSD (Labonté, Azoulay, Yerko, Turecki, & Brunet, 2014; Yehuda et al., 2015). Similarly, mothers with histories of interpersonal violence in their childhoods (i.e., physical and sexual abuse and exposure to intimate partner violence) and PTSD showed lower methylation at NR3C1 across CpGs that have been linked to adverse life events and related psychopathology and were associated with maladaptive interactions with their young children (Cordero et al., 2022). Further, it is likely that beyond the effects of early life exposure, later traumatic exposures in adulthood, the sex of the individual, as well as the persistence and treatment of psychiatric disorders further influence patterns of DNA methylation both within and individual and across generations.

Genome‐wide studies have also identified differential methylation patterns associated with childhood abuse (Suderman et al., 2014). Differences in DNAm are also detectable in response even to prenatal environmental factors. For example, peri‐conceptual exposures to famine and adversity resulted in differential methylation of several developmentally and immunologically active genes (Cao‐Lei et al., 2014; Tobi et al., 2009). Mothers' reports of adverse experiences during childhood were associated with DNAm age acceleration in male infants, providing support to the idea that DNAm age could reflect intergenerational biological embedding of mothers' childhood adversity (Dye et al., 2023). A longitudinal study examining DNAm patterns in adolescents found distinct differences linked to maternal stress during infancy and paternal stress during preschool years, as reported by parents (Essex et al., 2013). However, the outcomes associated with these DNAm differences remain to be fully understood.

Of course, the effects of early adversity are not uniform. Some individuals suffer devastating and persistent trauma‐related symptoms while others, with similar exposures, appear to thrive without any notable long‐term consequences. The relative risk and resilience of any given individual to early life adversity is likely due to the combination of both intrinsic and extrinsic factors, including factors only recently considered in the literature such as structural racism and historical trauma. Despite the ambiguity, it is evident that part of the individual variability is related to biological differences including genetic variation (e.g., diathesis stress, differential susceptibility), physiologic variability (e.g., biological sensitivity to context), and more complex combinations of genetic, developmental timing, and physiology, to some extent captured in the Adaptive Calibration Model (Del Giudice, Ellis, & Shirtcliff, 2011; Ellis & Del Giudice, 2014). Increasing evidence suggests that variability in developmental outcomes is also driven by epigenetic differences. Notably, models such as differential susceptibility and biological sensitivity to context propose that certain individuals are particularly sensitive to environmental influences, both positive and negative. These models hypothesize that individuals with specific genetic polymorphisms or patterns of physiological reactivity are not only more vulnerable to adverse experiences but also more capable of benefiting from positive experiences. For example, one study showed that the association between carrying a ‘risk’ allele of the FKBP5 gene and an increased risk of developing PTSD and depression in adults was mediated by DNAm changes occurring in response to childhood trauma. Specifically, childhood maltreatment was associated with reduced DNAm of the *FKBP5* gene, except in individuals homozygous for the protective allele (Klengel et al., 2013). Notably, these DNAm effects were specific to childhood trauma and not influenced by traumatic experiences in adulthood, suggesting that there may be a sensitive period during early development for the effects of epigenetic modification.

### The role of epigenetic changes due to early life adversity in later risk susceptibility

Differential sensitivity to the environment may also emerge due to early experiences that produce epigenetic changes that impact later sensitivity to the environment. For example, the altered patterns of DNAm in *NR3C1*, reported in both preclinical animal models and humans due to variability in early caregiving, have been hypothesized to contribute to elevated later risk for psychopathology due putatively to alterations in hippocampal‐dependent neuronal plasticity or through changes in the regulation of the HPA axis (Suderman et al., 2012). Additional support for the role of DNAm in early life shaping future responses to the environment comes from studies examining the impact of childhood maltreatment on the later development of psychopathology. Tyrka et al. (2015) examined maltreatment in children 3–5 years old. Assessing preschool children from low socioeconomic backgrounds, they found that maltreatment was associated with lower levels of *FKBP5* DNAm, which has been associated with reduced glucocorticoid receptor sensitivity, as well as higher levels of DNAm of *NR3C1* (Tyrka et al., 2015). The authors note that while the regulation of the HPA axis is complex and further studies are needed, *FKBP5* and *NR3C1* both influence regulation of the HPA axis and thus are potentially key genes altered by early adversity that influence risk for psychopathology through changes in HPA axis function (Parade et al., 2016; Tyrka et al., 2015). Parade et al. (2016) further investigated the potential links between *NR3C1* methylation and behavior problems in young children using the sample of preschool‐age children described above (see Tyrka et al., 2015). They found that DNAm of specific exons (coding regions of the gene) was associated with internalizing behavior and that DNAm mediated the relationship between early adversity and internalizing problems. This highlights the putative role of the HPA stress response system as it relates to the development of psychopathology in young children (Parade et al., 2016). Definitive data demonstrating concurrently, and ideally in the same biological source (e.g., blood, cell type, saliva, tissue), changes in DNAm, gene expression, cortisol reactivity, and behavioral measures are needed to confirm causal pathways between epigenetic changes, physiological differences, and psychopathology. To date, these critical studies have not appeared.

### The role of development and sensitive periods

When considering the implications of epigenetic modifications, such as DNAm in the brain, it is important to recognize there are developmental periods in which areas and circuits within the brain are uniquely susceptible to environmental influences. These sensitive periods are characterized by heightened neural plasticity (Knudsen, 2004). This developmental understanding has guided research in recent years to examine the association between when adverse experiences occur (e.g., early childhood) rather than considering childhood adversity as a binary construct (did vs. did not occur).

For instance, a study investigating children who experienced severe early‐life social deprivation in Romanian institutions found decreased DNAm of stress‐response‐related genes at age 12 years. These changes were directly associated with the proportion of time spent in institutional care during early development, highlighting the critical role of specific early‐life exposures in shaping epigenetic patterns (Non et al., 2016). For one of the genes examined, FKBP5, the strongest associations were found when the children were an average of 22 months of age, underscoring the importance of early rather than later exposure to adversity for this epigenetic marker. Conversely, associations with SLC6A4 were strongest at later time points, ranging from 30 months to 8 years of age. The distinct time frames at which each of these genes showed the strongest associations between the proportion of time the child had spent living within an institutional setting and epigenetic changes point to different sensitive periods for the two different genes (Non et al., 2016). Alterations in stress response genes found in this study were consistent with physiologic and behavioral outcomes of differences in cortisol reactivity and increased risk of psychopathology of this cohort at age 12 (Humphreys et al., 2015; McLaughlin et al., 2015). Behavioral data also support the idea of sensitive periods in development that are responsive to environmental input.

Additional studies have explored sensitive periods in early life when exposure to adversity is more strongly associated with differences in DNAm. A large, population‐based prospective birth cohort study found that the first 3 years of life appear to be a particularly sensitive period in which violence exposure elevates risk for externalizing symptoms when compared to children exposed at older ages, where the effect of violence emerges slowly over time until the age of 10 (Dunn et al., 2020). A prospective, longitudinal study using data from a large, population‐based sample of children found that exposure to adverse experiences (family instability, sexual/physical abuse, and caregiver physical/emotional abuse) between ages 3 and 5 predicted specific patterns of DNAm, mostly decreased, in children at 7 years, compared to exposures that occurred between ages 0–3 and 5–7 (Lussier et al., 2023).

Cicchetti and Handley (2017) also compared the impacts of various dimensions of childhood maltreatment, including the developmental timing of maltreatment, on the DNAm of the *NR3C1* at approximately 9 years of age. They found that children who experienced the onset of abuse or neglect during infancy or toddlerhood, but not children with onset in preschool years or beyond, showed significantly higher levels of *NR3C1* DNAm compared to nonmaltreated children. Greater chronicity and more subtypes of maltreatment experienced were also related to higher levels of DNAm, which in turn were related to psychological outcomes at a mean age of 9 years, including externalizing and depressive symptoms, emotional lability, and poor ego control (Cicchetti & Handley, 2017). Although significant gaps in the research remain, epigenetics has clearly emerged as a crucial process in the biological embedding of experiences and their relation to later risk for mental and physical illness. More understanding about the stability/malleability of molecular epigenetic processes will illuminate opportunities for prevention and remediation of adverse outcomes.

### Telomeres, adversity, and cellular aging

Another cellular marker of aging and development increasingly evaluated in relation to early life adversity and later health risk is telomere length (TL). Telomeres are a repetitive DNA sequence (TTAGGG in humans) – a cap on the ends of all eukaryotic chromosomes whose primary function is safeguarding genomic integrity during cell division by preventing the loss of genetic material. With each cell division, telomeres naturally shorten due to incomplete DNA replication. Eventually, when telomeres become critically short, cells can enter a state of senescence or undergo apoptosis, limiting further division (De Lange, 2005; Gilson & Géli, 2007; Greider, 1999). TL can also be affected by DNA damage, ionizing radiation, chronic inflammation, and exposure to early life stress. Beyond their role in cellular senescence, telomeres, including the associated RNA and protein components, also regulate gene expression across the genome through telomere position effects. Telomeres are key drivers of cellular differentiation, including neuronal differentiation, and overall genome stability. Fragments of telomeric DNA are even found floating outside of cells in extracellular vesicles serving as signalers in the surrounding tissues.

During embryonic development, telomeres elongate to ensure the proper functioning of rapidly dividing cells (Liu et al., 2007; Ozturk, Sozen, & Demir, 2014). Postnatally, telomere attrition occurs, but the rate and extent vary, influenced by developmental stage, genetic factors, environmental exposures, and psychosocial stressors. Unhealthy lifestyle choices, such as sedentary behavior and inadequate sleep, are associated with increased oxidative stress and inflammation, DNA damage, and shorter TL (Espinosa‐Otalora, Flórez‐Villamizar, Esteban‐Pérez, Forero‐Castro, & Marín‐Suarez, 2021). Additionally, in cross‐sectional studies, exposure to chronic stress, whether in early life or adulthood, has been linked to shorter TL (Coimbra, Carvalho, Moretti, Mello, & Belangero, 2017; Rentscher, Carroll, & Mitchell, 2020). Thus, TL is often considered a biological marker of cellular aging, reflecting the cumulative effects of environmental exposures, stressors, and genetic factors throughout an individual's life. Discovering how these factors influence TL in early life can provide insights into the mechanisms through which early adversity may impact cellular aging and disease risk.

Understanding telomere dynamics in early life holds significant importance due to its potential impact on lifelong health trajectories. The setting of telomere biology at birth, as highlighted by newborn TL studies, may have profound implications for an individual's lifelong health. The length of telomeres in childhood has emerged as a valuable predictive marker for various health outcomes in adulthood (e.g., Coimbra et al., 2017; Entringer et al., 2011; Martens et al., 2021), offering insights into the potential acceleration of cellular aging and an elevated vulnerability to age‐related diseases. Telomere attrition is significantly greater during the first years of life, with estimates of 200 base pair shortening annually compared to the 20–30 base pair annual loss in adults.

Similar to other epigenetic factors, there is wide variation in TL between individuals and, in some cases, the between‐individual differences are significantly larger than the TL change within an individual over time, creating challenges in characterizing change in TL over short periods and warranting careful consideration of sample size and expected effect size, particularly in light of differences in TL measurement precision across assays (Lin, Smith, Esteves, & Drury, 2019; Lindrose et al., 2021). Still, the accelerated rate of TL change early in life offers greater opportunity for the testing of TL in relation to early life experiences and exploration of the relation between the trajectory of TL change and future health outcomes. As with DNAm, it is plausible that the first years of life represent a unique sensitive period for the lasting effect of early life adversity on future health risk.

Moreover, the study of TL dynamics in early life may identify key windows of opportunity for interventions to prevent later risk. Understanding how early‐life stressors or favorable environmental influences impact telomere biology provides opportunities for targeted interventions aimed at promoting healthy development. This knowledge may contribute to designing strategies to mitigate the impact of adverse early‐life experiences on cellular aging and reduce the risk of associated health problems.

Recent research has made significant strides in understanding TL in infants, shedding light on the intricate factors influencing this critical aspect of cellular biology during early development. Among maternal influences, research indicates that higher maternal adverse childhood experiences (ACEs) correlate with shorter TL in infants, while telomere attrition from 4 to 18 months interacted with maternal ACEs to predict infant externalizing behaviors (Esteves et al., 2020). Additionally, increased symptoms of maternal depression were associated with shorter TL in infants TL at 18 months (Nelson, Allen, & Laurent, 2018). Higher levels of pregnancy‐specific stress also have been associated with shorter newborn TL, underscoring the impact of prenatal stressors on infant biology (Entringer et al., 2013; Marchetto et al., 2016; Send et al., 2017; Verner et al., 2021). Beyond maternal stress, in the study by Verner et al. (2021) although greater maternal stress predicted shorter newborn TL, higher maternal resilience, defined as positivity regressed on maternal stress, was associated with *longer* newborn TL, suggesting that attention to both the exposure to negative experiences during pregnancy and factors promoting positive well‐being together influence this critical biological ‘starting point’ for the next generations. Maternal nutrition during pregnancy has also been associated with newborn TL, with lower maternal folate levels – a key methyl donor – and higher triglyceride concentrations being significantly associated with shorter newborn TL (Entringer, Buss, & Wadhwa, 2012).

The shaping of TL extends beyond prenatal influences to include the early postnatal environment. For example, maternal depression during the first 3 years of life was found to predict shorter TL through the interaction with infant sex and temperament (Bosquet et al., 2024). Additionally, children with a greater amount of time living in a deprived institutional setting had significantly shorter TL than those who had less cumulative time before the age of 6 in the institutional setting (Drury et al., 2012). Within this same sample, TL measured repeatedly over time across early childhood shortened significantly more in children with a history of institutional caregiving compared to never‐institutionalized children (Humphreys et al., 2016). Evaluation of the relation between TL and psychiatric symptoms through age 16 indicated that shorter TL at 12 years predicted greater internalizing and overall symptoms of psychopathology and that the inverse pattern was not observed, highlighting a potential bidirectional relation between TL and risk (Wade, Fox, Zeanah, Nelson, & Drury, 2020).

Beyond the effects of early caregiving, neighborhood‐level disorder, poverty, violence, and maternal prenatal smoking have all been associated with shorter TL in children, highlighting the broader social determinants of health and biological aging from a young age (Drury et al., 2014, 2017; Esteves et al., 2020; Theall, Brett, Shirtcliff, Dunn, & Drury, 2013; Theall, McKasson, Mabile, Dunaway, & Drury, 2013). These findings suggest potential avenues for early intervention strategies to promote optimal cellular health in infancy and mitigate the impact of various maternal and environmental factors on telomere dynamics. Studying TL in infants has provided insights into the factors influencing telomere dynamics during early development and illuminated the long‐term implications of early telomere biology as, over the life‐span, there is significant rank‐order TL stability – meaning that individuals with shorter TL early in life are far more likely to have shorter TL in adulthood (Martens et al., 2021). Research has shown shorter telomeres have been associated with conditions such as obesity, cardiovascular diseases, cancer, and neurodegenerative disorders. Furthermore, studies have linked TL at early ages to behavioral outcomes (e.g., ADHD; Pham et al., 2022), suggesting that telomere biology may play a role in shaping brain development and influencing behavioral health during critical periods of early childhood. The intricate interplay between telomeres and the risk of these health outcomes underscores the significance of understanding telomere dynamics for immediate health assessments and predicting and mitigating long‐term health risks.

### Inflammation

Inflammation encompasses a complex set of biological responses initiated as a protective mechanism against harmful stimuli, such as infectious agents, damaged cells, or irritants. The regulation of inflammation is tightly controlled, involving intricate molecular pathways and feedback mechanisms to ensure an appropriate response to different challenges while avoiding excessive or prolonged activation. Immune responses involve various immune cells, signaling molecules, and vascular changes, and, like other stress‐responsive systems, exhibit differences in immune responses across development. Activated immune cells release signaling molecules like cytokines, chemokines, and prostaglandins. These, in turn, recruit more immune cells to the site of injury or infection, which attempt to neutralize and eliminate the source of threat, and tissue repair processes are initiated. Inflammatory processes are designed to eliminate cellular injury, clear out damaged cells and tissues, and initiate tissue repair. Although acute inflammation provides an essential defense for maintaining tissue homeostasis, chronic inflammation can be detrimental and is associated with a variety of health conditions, including autoimmune diseases, allergies, chronic illnesses, and psychiatric disorders (Liu et al., 2017). Over the last decade, chronic low‐grade immune activation and/or repeated activation of the immune system are increasingly being recognized as another pathway tied to accelerated aging linked to the same age‐related illness predicted by ACEs (Franceschi, Garagnani, Parini, Giuliani, & Santoro, 2018).

Mounting evidence suggests intricate connections between systemic inflammation and the central nervous system that influence mood, cognition, and mental well‐being. Chronic inflammation has been implicated in the pathophysiology of psychiatric disorders such as depression, anxiety, and schizophrenia, and inflammation early in life associated with adverse childhood experiences has been linked to an elevated risk of mental health disorders later in life (Réus et al., 2015). Elevated levels of inflammatory markers, such as C‐reactive protein (CRP) and proinflammatory cytokines, have been associated with an increased risk of developing mood disorders (Milton et al., 2021). Moreover, the bidirectional communication between the immune system and the brain, known as the immune–brain axis, plays a crucial role in shaping behavioral responses to stress and emotional regulation (Jin, Li, Jeong, Castro‐Martinez, & Zuker, 2024). Understanding intricate links between inflammation and psychiatric disorders contributes to elucidating underlying mechanisms through which experiences become biologically embedded and provide opportunities to develop interventions that target the immune and nervous systems in order to promote health (O'Connor et al., 2020).

Studying inflammation during the early years is essential due to its profound implications for lifelong health and development. Early‐life inflammation can exert long‐lasting effects on various physiological systems, influencing the trajectory of immune function, neurodevelopment, and overall well‐being. Investigating inflammatory processes during infancy provides crucial insights into the interplay between environmental exposures, genetic factors, and the developing immune system. Additionally, understanding how inflammatory processes unfold in early life is important to elucidate potential links to later physical and mental health outcomes, including cardiovascular diseases, autoimmune conditions, and neurodevelopmental disorders (Jiang, Cowan, Moonah, & Petri, 2018; Jin et al., 2024). Early‐life inflammation is increasingly recognized as a critical factor in shaping long‐term health trajectories, emphasizing the need for comprehensive research to inform preventive strategies and interventions.

The literature on inflammation and infancy reveals a complex interplay between early immune responses and subsequent health outcomes. For example, research has shown that lower socioeconomic level and higher maternal psychosocial stress independently predict CRP elevations in 17‐month‐old infants (David, Measelle, Ostlund, & Ablow, 2017). Other research also has demonstrated the impact of caregiving by comparing children in foster care and children with their biological parents, aged 24 months to 7 years. The results demonstrated that caregiving quality influenced cortisol/DHEA ratios (a measure of chronic stress and HPA axis function) and secretory IgA concentrations (Reindl et al., 2022). Specifically, children in foster care with lower caregiving quality had lower cortisol/DHEA ratios compared to those with higher caregiving quality. Additionally, they showed a decline, rather than an increase, in secretory IgA concentrations over the study period. The authors speculated that since secretory IgA spikes in response to stress but then declines, this might explain the decreasing secretory IgA levels found in children in foster care of lower caregiving quality across the study period, while children in foster care of higher caregiving quality may have expected age‐related increases.

Recent literature also indicates that early exposure to inflammation can profoundly influence neurodevelopment and emotional health. Studies have demonstrated associations between elevated inflammatory markers in early childhood and internalizing and externalizing subsequent problems (Pham et al., 2022). A large prospective observational study in Bangladesh (Jiang et al., 2014) directly assessed the association of biomarkers of inflammation at 6 months of age on motor and cognitive function at 12 and 24 months. Fever and inflammation were strongly associated with lower language, cognitive, and motor test scores. Understanding the interplay of immune and stress response factors is essential for developing interventions and strategies to promote healthy immune development and mitigate the risk of inflammatory‐related health issues during infancy and beyond. Similar to epigenetic processes and telomere regulation, inflammation also can be measured in serum or plasma, and additional research is needed to determine how that may influence study outcomes.

### Interrelations among these processes

Although DNA methylation, telomere regulation, and inflammatory processes exert unique influences on behavior and predisposition to disease states, they also are interrelated (Zhu et al., 2021). DNAm patterns can impact TL, influencing the rate of attrition or maintenance. In turn, telomeres are sensitive to environmental factors, including epigenetic modifications like DNA methylation (Adwan‐Shekhidem & Atzmon, 2018; Buxton et al., 2014; Dong et al., 2017). DNAm patterns established early also can influence the expression of genes associated with inflammation and cellular aging (Aquino et al., 2018; Jones, Goodman, & Kobor, 2015; McDade et al., 2017). Inflammation, as a response to various environmental stimuli, is closely linked to both DNAm and telomere dynamics. Inflammatory processes can contribute to accelerated telomere shortening (Barnes, Fouquerel, & Opresko, 2019), while, conversely, shorter telomeres may exacerbate inflammatory responses (Zhang et al., 2016).

Oxidative stress is another measurable biological process associated with aging, stress, and both telomere shortening and inflammation. Oxidative stress is elevated in certain inflammatory states, particularly proinflammatory states, and is also a significant predictor of telomere shortening through the effects of nitric oxide and the production of reactive oxygen species, and subsequent DNA damage. Future studies are needed to evaluate the interaction of these pathways as potential mechanisms underlying the established links between early life adversity, aging‐related health conditions, and psychopathology (see Alameda et al., 2018). Alterations to the inflammatory pathways, in addition to established links to mental illness, are also implicated in physical health outcomes, including cardiovascular disease, obesity, and diabetes – all negative physical health consequences associated with early life adversity.

Understanding these interrelationships in early development is crucial for unraveling the early determinants of accelerated biological aging and the link to later health outcomes. It provides insights into how the epigenome, telomeres, and inflammatory pathways converge to shape the developmental trajectory and set the stage for potential health implications later in life.

## Clinical implications

Clearly, understanding cellular and molecular processes at the level of an individual may not soon be a part of the repertoire of infant mental health practitioners. Nevertheless, awareness of advances in research that may enhance the selection and effectiveness of treatment for individual children is of great interest for our field. Medicine in general is in the early stages of moving from gold standard treatments to patient‐specific treatments as we enter a new era of precision medicine (National Research Council, 2011). At this point, precision medicine is focused largely on genomic‐driven precision in pharmacology (Akhoon, 2021), but it is likely that psychotherapy and dyadic interventions, known to be evidence‐based, also involve changes to biological processes similar to those induced by pharmacology. As a well‐known psychopharmacologist has noted, psychotherapy can be considered ‘as a neurobiological probe capable of inducing epigenetic changes in brain circuits not unlike the ultimate actions of psychotropic drugs’ (Stahl, 2012, p. 251). Beyond this, given the existing evidence that early adversity results in biological changes at the molecular level, a critical next step to ensuring healthy lifelong trajectories for children exposed to early life adversity is to determine the effect of existing evidence‐based interventions on more than just caregiver‐reported symptoms. Specifically, studies should evaluate whether current treatments presumed effective for behavior and emotions are equally impactful for correcting alterations in biologic pathways (e.g., DNA methylation, telomere length, levels of reactive oxygen species, inflammation). It may well be that, in the not‐so‐distant future, interventions for young children will be selected based on baseline biological profiles of individuals and outcomes measured both by caregiver report and through measurement of biological pathways.

Already, molecular effects of psychotherapy with young children have been demonstrated. In a small intervention study, 23 infants aged 6–21 months were randomized to receive attachment and biobehavioral catch‐up (ABC) or a control intervention. DNAm was comparable in the two groups preintervention, but DNAm varied between the ABC and the control group at 14,828 sites postintervention. These differences were associated with genes involved in cell signaling, metabolism, and neuronal development (Hoye et al., 2020). Further, a recent study of 2‐ to 6‐year‐old low‐income children who had experienced trauma and who received treatment with child parent psychotherapy had epigenetic aging assessed at baseline and posttreatment (Sullivan et al., 2024). Using a quasi‐experimental design with a propensity matched control sample, they found the two groups had similar epigenetic ages at baseline. After treatment, however, epigenetic age acceleration in the treatment group was reduced compared to the matched community sample. This is the first demonstration that a relationship‐based intervention in young children ameliorates accelerated biological aging in traumatized young children. Unfortunately, the authors did not report whether symptomatic improvement converged with or diverged from the epigenetic aging.

On the other hand, Braithwaite et al. (2023) reported results from an RCT comparing Video Intervention to Promote Positive Parenting and Sensitive Discipline to primary caregivers of 12‐ to 36‐month‐olds. They used buccal swabs to assess DNAm of three genes – *NR3C1*, *FKBP5*, and *OXYR*. They found no group differences in DNAm of *NR3C1* or *OXYR* genes and no associations of DNAm with child behavior. They did find a significant interaction between group and sex, such that boys in the care as usual group had higher DNAm of *FKPB5* than girls, but in the intervention group, girls had higher DNAm of *FKBP5* than boys.

These results highlight the complexity of the processes involved and remind us that the measures may not always be affected consistently. The variability in results in these preliminary studies may derive from differences in assays, populations, and/or interventions being studied.

Beyond these emerging studies, there are several additional areas in which research advances in cell and molecular biology may be especially important to infant mental health practitioners. Specificity of treatment, prevention, and physical health are three areas in which to anticipate contributions.

First, with an increasing array of evidence‐based infant mental health interventions at our disposal, a critical yet largely unanswered question for our field is: what interventions are effective for whom? Even with treatments that have demonstrated effectiveness, not all individuals exhibit a positive response, and for some, the response may be only partial. For example, estimates are that 30%–40% of adults receiving psychotherapy are not responsive to treatment (Gloster et al., 2020). Dropout rates also are sometimes quite high. In a study of 1,240 children and adolescents who received trauma‐focused cognitive behavioral therapy, 24% were nonresponders and 13% dropped out (Skar et al., 2022). This highlights an area where a deeper understanding of molecular pathways could offer opportunities to tailor treatments to individual needs and optimize outcomes.

Second, prevention is always a fundamental component of infant mental health interventions due to the brain's remarkable plasticity in the earliest years of life and the crucial role of caregiving relationships in shaping young children's developmental paths. A better understanding of cellular and molecular mechanisms may shed more light on individuals at risk for mental health challenges stemming from various forms of early adverse experiences, even before symptoms manifest. By elucidating these processes, we might enhance our ability to prevent long‐term distress and disability resulting from psychiatric disorders. In essence, rather than relying on broadly defined risk factors, we could potentially identify with greater precision who is more vulnerable to adverse outcomes and the underlying reasons for this susceptibility.

Third, progress in our understanding of cellular and molecular processes can be harnessed to prevent physical morbidity and mortality. As illustrated by the ACE study, adverse childhood experiences affect physical as well as mental health. According to the weathering hypothesis proposed by Geronimus (1992), chronic exposure to social and economic adversity, including racism, may accelerate the deterioration of an individual's physical health, contributing to well‐documented racial disparities in health outcomes (Geronimus, 1992). A systematic review of 41 studies found support for the weathering hypothesis, though there was variability in the strength of the association with various disease states (Forde, Crookes, Suglia, & Demmer, 2019). Growing evidence suggests that adverse experiences influence cellular and molecular processes involved in the onset of disease states. Preventing health problems, even in the absence of emotional and behavioral abnormalities, could potentially expand the future role of infant mental health practitioners.

## Conclusions

Although we are only beginning to appreciate the cellular and molecular processes that are involved in the biological embedding of experience, advances are likely to increase exponentially in the next two decades. This illustrative review cannot begin to cover all the relevant cell and molecular mechanisms that are involved in risk and resilience. Nevertheless, we selected epigenetics, telomere regulation, and inflammation as examples of active areas of investigation relevant to the goal of identifying early indicators of adversity and recovery from adversity. Because infant mental health practice is focused on the earliest years of life, its practitioners are well positioned to direct resources and design experiences that may in the future target corrective processes, including at the cellular and molecular level.

## Ethical considerations

As a review article, no human subjects issues were involved in its preparation.

**Key points**

**What's known?**

- Biological embedding of adverse experiences involves cellular and molecular processes that we are beginning to understand.

**What's new?**

- DNA methylation, telomere regulation, and inflammation are three active areas of research that have begun to explore the relation of adverse experiences to subsequent health and mental health outcomes.
- These advances hold promise to enhance the precision of interventions, prevention of later emerging disorders and diseases, and an expanded role for mental health practitioners to focus on prevention of physical as well as mental health disturbances.

**What's relevant?**

- Because infant mental health practice is focused on the earliest years of life, its practitioners are well positioned to direct resources and design experiences that may in the future target corrective processes, including at the cellular and molecular levels.

## Data Availability

Data sharing not applicable to this article as no datasets were generated or analyzed during the current study.
